# Effect of cryopreservation on proliferation and differentiation of periodontal ligament stem cell sheets

**DOI:** 10.1186/s13287-017-0530-5

**Published:** 2017-04-17

**Authors:** Mengying Li, Cheng Feng, Xiuge Gu, Qin He, Fulan Wei

**Affiliations:** 1 Department of Orthodontics, School of Stomatology, Shandong University, Jinan, People’s Republic of China; 2 Shandong Provincial Key Laboratory of Oral Tissue Regeneration, School of Stomatology, Shandong University, Wenhua Xi Road No. 44-1, Jinan, Shandong 250012 People’s Republic of China; 3 Jinan Hospital of Traditional Chinese Medicine, Jinan, People’s Republic of China

**Keywords:** Cryopreservation, Periodontal ligament stem cell, Cell sheet

## Abstract

**Background:**

Cryopreservation has been extensively applied to the long-term storage of a diverse range of biological materials. However, no comprehensive study is currently available on the cryopreservation of periodontal ligament stem cell (PDLSC) sheets which have been suggested as excellent transplant materials for periodontal tissue regeneration. The aim of this study is to investigate the effect of cryopreservation on the structural integrity and functional viability of PDLSC sheets.

**Methods:**

PDLSC sheets prepared from extracted human molars were divided into two groups: the cryopreservation group (cPDLSC sheets) and the freshly prepared control group (fPDLSC sheets). The cPDLSC sheets were cryopreserved in a solution consisting of 90% fetal bovine serum and 10% dimethyl sulfoxide for 3 months. Cell viability and cell proliferation rates of PDLSCs in both groups were evaluated by cell viability assay and 3-(4,5-dimethylthiazol-2-yl)-2,5-diphenyltetrazolium bromide (MTT) assay, respectively. The multilineage differentiation potentials of the cells were assessed by von Kossa staining and Oil Red O staining. The chromosomal stability was examined by karyotype analysis. Moreover, the cell sheets in each group were transplanted subcutaneously into the dorsal site of nude mice, after which Sirius Red staining was performed to analyze the efficiency of tissue regeneration.

**Results:**

The PDLSCs derived from both groups of cell sheets showed no significant difference in their viability, proliferative capacities, and multilineage differentiation potentials, as well as chromosomal stability. Furthermore, transplantation experiments based on a mouse model demonstrated that the cPDLSC sheets were equally effective in generating viable osteoid tissues in vivo as their freshly prepared counterparts. In both cases, the regenerated tissues showed similar network patterns of bone-like matrix.

**Conclusions:**

Our results offer convincing evidence that cryopreservation does not alter the biological properties of PDLSC sheets and could enhance their clinical utility in tissue regeneration.

## Background

Tissue engineering based on biocompatible and biodegradable scaffolds has emerged as an attractive field in regenerative medicine over the past several decades [1]. Despite recent advances, clinical application of implantable scaffolds remains limited due to several major drawbacks, including insufficient cell proliferation and adhesion, undesirable stimulation of the local inflammatory response, difficulty in balancing cell proliferation with scaffold degradation, and the inability to generate functionally competent tissues [2]. To address these limitations, cell sheet engineering, in which one or multiple layers of intact cell sheets are grown on and subsequently detached from a thermosensitive surface without the use of a scaffold, has been developed as an alternative approach for tissue regeneration. Compared to the scaffold-based approach, cell sheet engineering offers the obvious advantage of well-preserved endogenous extracellular matrix (ECM) and cellular junctions which greatly increases the chances of success in transplantation [3]. Cell sheet engineering has been applied to the construction of soft and hard tissues alike, including myocardial tissues [4, 5], liver tissues [6], bone [7], and blood vessels [8], etc. Previously, we reported the development of an effective and reliable method based on vitamin C (Vc) treatment that facilitated the construction of highly viable and functional periodontal ligament stem cell (PDLSC) sheets and the subsequent regeneration of periodontal tissues [9]. Vc has been shown to promote the generation of collagen and other ECM constituents [10–12], as well as to mimic the in vivo physiological environment. Besides, when supplied to the culture medium, it can act as a growth promoter to stimulate cell proliferation [13]. We then successfully employed PDLSC sheets to regenerate a functional bio-root structure for artificial crown restoration [14]. However, clinical application of PDLSC sheets was limited by the fact that their laboratory preparation was very time consuming. In fact, using conventional methods, it would require at least 10 days to grow the cell sheets in our laboratory which precluded any therapeutic usage in the event of medical emergency.

Cryopreservation has been extensively studied as a viable solution to the long-term storage of various biomaterials, such as oocytes [15], stem cells [16, 17], vascular tissues [18], and embryos [19]. Recently, several groups demonstrated the feasibility of obtaining viable PDLSCs from frozen periodontal ligament tissues or intact whole teeth [20, 21]. However, to the best of our knowledge there has been no comprehensive study on the impact of cryopreservation on PDLSC sheets. To this end, our current study aims to investigate whether cryopreservation could affect the structural integrity and physiological function of PDLSC sheets. We also compared the proliferative capacities and differentiation potentials of PDLSCs derived from cryopreserved or freshly prepared cell sheets. Finally, we examined whether the cryopreserved PDLSC sheets could be used as implants for tissue regeneration in a mouse model.

## Methods

### Cell culture

All protocols for the handling of human tissues were approved by the Research Ethics Committee of Shandong University (No. MECSDUMS2012087). Informed consent was obtained from the donors and their parents. The animal study was reviewed and approved by the Committee on the Ethics of Animal Experiments of Shandong University (No. ECAESDUSM2012075).

Extracted human impacted third molars were collected from 16 subjects being treated at the Department of Oral and Maxillofacial Surgery, Stomatological Hospital of Shandong University. Human PDLSCs were isolated from the root surface and digested in a solution of 3 mg/mL collagenase type I (Sigma-Aldrich, USA) and 4 mg/mL dispase (Sigma-Aldrich) for 1 h at 37 °C as previously reported [22]. PDLSCs were cultured at 37 °C under 5% carbon dioxide in 25 cm^2^ flasks (Corning, USA) using alpha-modified Eagle’s medium (α-MEM; Invitrogen, USA) supplemented with 15% fetal bovine serum (FBS; Invitrogen), 2 mmol/L glutamine, 100 U/mL penicillin, and 100 μg/mL streptomycin (Invitrogen). Cells were then passaged two to three times in the same medium before being used for the growth of cell sheets.

### Preparation of PDLSC sheets

To induce the growth of cell sheets, PDLSCs were cultured in 60-mm culture dishes (Corning, USA) with Vc (Sigma-Aldrich) added to the PDLSC culture to a final concentration of 20 μg/mL [9]. After reaching confluency in 2–3 days, the PDLSCs were cultivated for an additional 10–14 days until the cells at the edge of the dishes started to wrap up, indicating the formation of cell sheets. The intact sheets were isolated with a blunt blade under humidified conditions when they reached an average thickness of around two layers of cells. The cell sheet will then shrink to about 15 mm due to the elasticity. The isolated cell sheets were assigned to the cryopreservation group (cPDLSC) or the control group (freshly prepared PDLSC; fPDLSC) for the following experiments. Paired cell sheets (cPDLSC sheet and fPDLSC sheet) were prepared from molars of the same subject. The cPDLSC sheets were submitted to the cryopreservation and thawing procedures described below, whereas the ones in the control group were freshly prepared and directly used for subsequent studies.

### Cryopresevation and thawing of PDLSC sheets

All experiment procedures pertaining to the cryopreservation and recovery of cell sheets were conducted under a sterile environment, either on a clean bench or with the containers wrapped in parafilm (Bemis® Flexible Packaging) to prevent contamination.

For cryopreservation, the cell sheets were directly equilibrated in a pre-chilled solution mix of 90% FBS and 10% dimethyl sulfoxide (DMSO; Sigma-Aldrich) at 4 °C. The subsequent chilling and freezing was performed in a controlled manner using a programmable freezer (Taiyo-Toyo Sanso Co., Japan). The temperature drop rate was –0.5 °C /min from 4 °C to –20 °C, and –1 °C /min from –20 °C to –80 °C. After 24 h incubation at –80 °C, the cryovials were submerged in liquid nitrogen. Woods et al. reported that differences in viability were not statistically significant comparing 1 week to 1 month to 6 months [23]; in the present study, we stored the sheets in liquid nitrogen for 3 months.

For thawing, the cryovials were retrieved from the liquid nitrogen, rapidly immersed in a 37 °C water bath and gently agitated until the cryopreservation medium was completely melted. The cell sheet was then transferred to tubes containing 5 mL α-MEM supplemented with 15% FBS. The tubes were placed into the shaker, gently agitated at 1000 rpm for 5 min at 37 °C, and then centrifuged at 1000 rpm for 5 min. After the supernatant was discarded, the pelleted cell sheets were submitted to the same procedures mentioned above. Finally, the cell sheets were carefully transferred to culture plates each containing 5 mL of the same culture medium as described above and cultivated at 37 °C under 5% CO_2_.

### Immunohistochemistry of cryopreserved PDLSC sheets

A series of 5-μm thick cryosections of cPDLSC sheets were prepared and incubated at room temperature for 60 min in 50 mM Tris-buffered saline with 0.4% Triton X-100 (TBS-T; pH 7.2) containing 5% bovine serum albumin (BSA). For the detection of fibronectin, the cells were stained overnight with 1:500 anti-fibronectin (Sigma-Aldrich) diluted in TBS-T containing 1% BSA. After washing to remove the unbound primary antibodies, the cells were then incubated at room temperature for 1 h with 1:200 fluorescein isothiocyanate (FITC)-labeled goat anti-mouse immunoglobulin M (Chemicon, USA) diluted in TBS-T containing 1% BSA. For the detection of integrin, 1:500 anti-integrin (Sigma-Aldrich) and 1:200 FITC-labeled goat anti-mouse immunoglobulin M (Chemicon) were used as the primary and secondary antibodies, respectively. For the detection of collagen type I, 1:1000 anti-collagen type I (Sigma-Aldrich) and 1:200 phycoerythrin-labeled goat anti-mouse immunoglobulin G (Chemicon) were used as the primary and secondary antibodies, respectively. The fluorescently labeled cell sheets were viewed under a confocal laser scanning microscope (Carl Zeiss LSM700, Germany).

### Cell viability assay

After thawing, the viability of the PDLSC sheets was assessed by the LIVE/DEAD Viability/Cytotoxicity Kit (Invitrogen). Briefly, the cell sheets were washed with phosphate-buffered saline (PBS) and then the cell sheets were mechanically disrupted, followed by incubation for 45 min at room temperature in 150 μL combined Live/Dead solution provided in the kit. Imaging was performed using a microscope (Olympus IX71, Japan) at 10× magnification. Ten fields of microscope were used for statistics of the cell viability rate. Live cells emitted green fluorescence due to the cleavage of membrane-permeated calcein AM by intracellular esterases, whereas dead cells were characterized by their emission of red fluorescence generated from the labeling of nucleic acids by membrane-impermeable ethidium homodimer-1.

### 3-(4,5-dimethylthiazol-2-yl)-2,5-diphenyltetrazolium bromide (MTT) assay

PDLSC sheets were mechanically disrupted. The mechanically disrupted cells and the fresh PDLSCs (fPDLSCs) were seeded into 96-well plates at a cell density of 1 × 10^4^ cells/mL, followed by cultivation in α-MEM with 15% FBS at 37 °C under 5% carbon dioxide for 24 h, 48 h, and 72 h, respectively. Subsequently, the culture medium was replaced with 5 mg/mL of MTT solution (Sigma-Aldrich) diluted in PBS. The plates were incubated again for 4 h at 37 °C and a volume of 150 μL DMSO was added to each well. The plates were then agitated for 10 min to ensure the dissolution of any remaining crystals. Optical density (OD) was measured at 490 nm (*A*_490_).

### Multilineage differentiation potential

fPDLSC sheets and cPDLSC sheets were disrupted and the resultant cells were separately incubated in osteo-inductive medium (complete medium supplemented with 50 mg/L Vc, 10 nmol/L dexamethasone, and 10 mmol/L β-glycerophosphate) and adipo-inductive medium (0.5 mmol/L isobutyl-methylxanthine, 60 μmol/L indometacin, 0.5 μmol/L hydrocortisone, and 10 μg/mL insulin) for 3 weeks. Subsequently, the cells were separately stained with von Kossa and Oil Red O. The control groups were cultivated in α-MEM supplemented with FBS, penicillin and streptomycin. For Von Kossa, samples were washed with PBS, fixed in 4% paraformaldehyde, treated with 1% silver nitrate solution, and incubated under ultraviolet light for 10 min. Wells were washed with sodium thiosulfate overnight and imaged under transmitted light. For the Oil Red O reagent, stock solutions were prepared using 3 mg/mL Oil Red O (O-0625; Sigma-Aldrich) in isopropanol. Working solutions consisted of 3:2 Oil Red O stock in deionized water. For staining, cells were fixed in 4% paraformaldehyde, washed, and treated with 60% ethanol for 5 min. Oil Red O stain was added for 5 min, and samples were imaged for fat droplet formation, with droplets appearing red under transmitted light. The expression levels of runt-related transcription factor 2 (RUNX2), osterix (OSX), osteocalcin (OCN), peroxisome proliferating activated receptor γ (PPARγ2), and lipoprotein lipase (LPL) were measured by reverse transcription polymerase chain reaction (RT-PCR) to evaluate the extent of osteogenic differentiation and adipogenic differentiation. The gene primers used in this study are listed in Table 1. Total RNA isolation, first-strand cDNA synthesis, and PCR reactions were performed as described previously [9].

**Table 1:** List of primers used in real time polymerase chain reaction

Primer ID	Sequence (5′–3′)
RUNX2-F	GTTTCACCTTGACCATAACCGT
RUNX2-R	GGGACACCTACTCTCATACTGG
OSX-F	ACCTACCCATCTGACTTTGCTC
OSX-R	CCACTATTTCCCACTGCCTTG
OCN-F	AATCCGGACTGTGACGAGTTG
OCN-R	CAGCAGAGCGACACCCTAGAC
PPARγ2-F	CTCCTATTGACCCAGAAAGC
PPARγ2-R	GTAGAGCTGAGTCTTCTCAG
LPL-F	ATGGAGAGCAAAGCCCTGCTC
LPL-R	GTTAGGTCCAGCTGGATCGAG
GAPDH-F	TGGGCAAGATTAAGATCGGAAT
GAPDH-R	TTGATGTCGCTGTGCTTCCA

*GAPDH* glyceraldehyde 3-phosphate dehydrogenase, *F* forward, *LPL* lipoprotein lipase, *OCN* osteocalcin, *OSX* osterix, *PPARγ2* peroxisome proliferating activated receptor γ, *R* reverse, *RUNX2* runt-related transcription factor 2

### Karyotype analysis

G-banded karyotype analysis was employed to examine the chromosomal stability of cPDLSC and fPDLSC sheets. Preparation of chromosomes was performed using a previously described protocol with minor modifications [24]. In brief, the cell sheets were disrupted and the resultant cells were detached by being treated with a final concentration of 0.2 μL/mL colcemid for 2.5 h. Then, 8 mL hypotonic solution containing 0.075 M KCl was added and the resulting cell suspension was incubated for 30 min at 37 °C. The cells were then fixed using methanol-acetic acid (3:1) fixative and centrifuged at 1500 rpm for 10 min. The fixation experiment and the subsequent centrifugation was performed a total of three times. The pellet was re-suspended in fresh fixative (as above) and spread on slides. A drop of the suspension was carefully placed onto a wet microscope slide and allowed to dry under moderate humidity (around 50%). Giemsa banding (GTG-banding) was also performed using a previously described protocol with minor modifications [24]. In brief, slides were incubated in trypsin solution for 8–10 s, rinsed in normal saline (sodium chloride 0.9%) three times, then stained in 10% Giemsa stain (Sigma-Aldrich) in phosphate buffer (pH 6.8) for 1.5 min. Slides were then rinsed in phosphate buffer (pH 6.8) three times, dried, and mounted in Entellan mountant (Sigma-Aldrich). The chromosome number, chromosome length, kinetochore position, and G-band position were observed according to the ISCN 2005 standard [25].

### Animal model and transplantation of cell sheets

A total of 24 female nude mice were raised under specific pathogen-free conditions up to 5 weeks. The mice were then anesthetized via an intraperitoneal injection of 10% chloral hydrate at a dose of 0.003 mL/g body weight. Nude mice were randomly assigned to two experiment groups that were transplanted with cPDLSC and fPDLSC sheets, respectively. The prepared cell sheets were transplanted subcutaneously into the dorsal site of nude mice as previously described [9]. All animals were sacrificed 4 weeks after transplantation. The regenerated tissue samples were isolated, fixed with 4% paraformaldehyde, and subjected to histological examinations.

### Sirius Red staining

Sirius Red staining was conducted as previously described [26]. Briefly, the tissue sections were dewaxed, dipped into water, stained with 1 g/L Picric Acid-Sirius Red at 37 °C for 1 h, and then washed with water. The sections were mounted and viewed under a polarized light microscope (Leica DM5000 B, Germany) and darkfield images were obtained.

### Statistical analysis

Student’s *t* test was used to analyze the differences between experimental groups. *P* < 0.05 was considered statistically significant. All experiments were performed in triplicate.

## Results

### The ECM in cPDLSC sheets was not disrupted by freezing or thawing

**Fig. 1:**
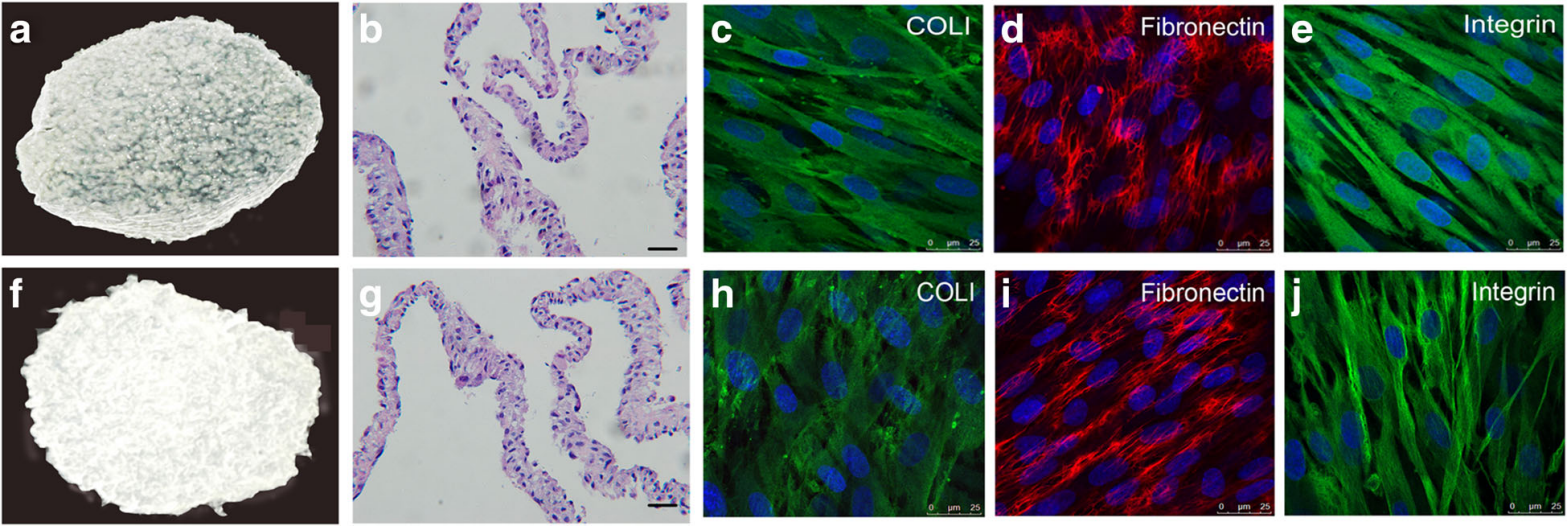
The ECM was intact and undisrupted in cPDLSC sheets. Macroscope of cPDLSC sheet (**a**) and fPDLSC sheet (**f**) shows that their “sheet-like” structures were intact. H&E staining of cPDLSC sheets (**b**) and fPDLSC sheets (**g**) showing a uniform and multilayer tissue structure. Visualization of type I collagen (*COLI*; **c**), fibronectin (**d**), and integrin (**e**) in cPDLSC sheets by immunostaining. Visualization of type I collagen (**h**), fibronectin (**i**), and integrin (**j**) in fPDLSC sheets by immunostaining. *Scale bars* = (**b**,**g**) 20 μm; (**c**–**e**, **h**–**j**) 25 μm. Magnification: (**b**, **g**) 40×; (**c**–**e**, **h**–**j**) 100 ×

We began our study by investigating whether the cryopreservation method that we used had any detrimental effects on the vital structural organization of PDLSC sheets. Both cPDLSC and fPDLSC sheets, which were induced only by α-MEM supplemented with 15% FBS and 20 μg/mL Vc, retained their “sheet-like” structure (Fig. 1a and f) with the ECM remaining largely intact (Fig. 1c–e and h–j). Subsequent hematoxylin and eosin (H&E) staining of both types of sheets showed a uniform, two-dimensional tissue structure consisting of two to three layers (Fig. 1b and g). To ascertain the structural integrity of the PDLSC sheets, immunostaining was performed to determine the distribution of several main ECM components, including fibronectin, type I collagen, and integrin. The results showed that all three types of matrix proteins were abundantly distributed around the PDLSCs in both the cryopreserved sheets (Fig. 1c–e) and in their freshly prepared counterparts (Fig. 1h–j), implying that the ECM structure remained intact and undisrupted despite cryopreservation.

### Cryopreservation and thawing had no detectable impact on the viability of PDLSCs

**Fig. 2:**
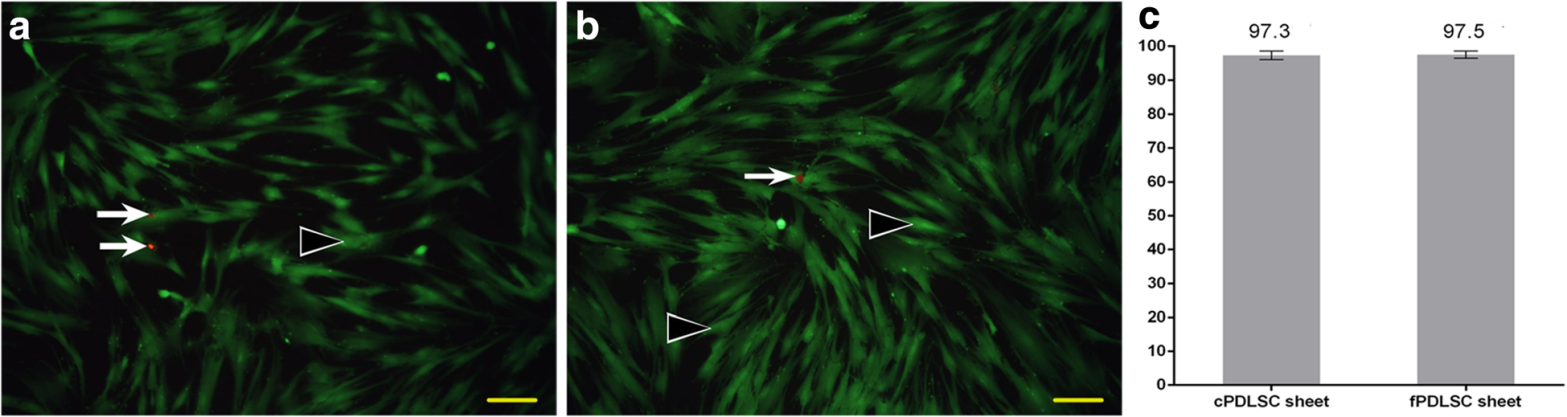
The cryopreserved periodontal ligament stem cell (*cPDLSC*; **a**) and freshly prepared periodontal ligament stem cell (*fPDLSC*; **b**) sheets had similar cell viability rates as indicated by the EdU-based cell viability assay. Examples of live and dead cells are indicated in *green* and *red*, respectively (*solid triangles*: live cells; *arrows*: dead cells). **c** Different cell viability rates of fPDLSC sheets (97.5%) and cPDLSC sheets (97.3%). *P* = 0.7425. *Scale bars* = 40 μm. Magnification: 20×

The functional viability of cells in both cPDLSC and fPDLSC sheets was assessed by cell viability assay. About 2.5 × 10^6^ cells were observed per sheet. No significant difference in the number of live or dead cells was observed between cPDLSC and fPDLSC sheets (Fig. 2a and b). Cell viability rates of the two groups are out of statistical significance (*P* > 0.05; Fig. 2c), indicating that neither cryopreservation nor thawing had a significant impact on cell viability.

### Cryopreservation and thawing did not negatively affect the proliferative capacities of PDLSCs

**Fig. 3:**
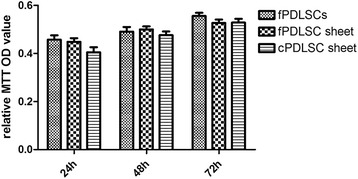
Determination of cell proliferative potential by MTT assay. The periodontal ligament stem cells (PDLSCs) from fresh sheets (*fPDLSC sheet*) have a similar proliferation rate when compared to fresh PDLSCs (*fPDLSCs*) at 24 h. PDLSCs derived from the cryopreserved sheets (*cPDLSC sheet*) exhibited a slightly slower proliferation rate compared to those from the freshly prepared sheets and fPDLSCs in the first 24 h following the inoculation in α-MEM. However, the three groups of cells showed roughly the same proliferative potential at 48 and 72 h. *P* =0.8055, fPDLSC versus fPDLSC sheet; *P* =0.5281, fPDLSC versus cPDLSC sheet; *P* =0.6313, fPDLSC sheet versus cPDLSC sheet. All *P* > 0.05. MTT 3-(4,5-dimethylthiazol-2-yl)-2,5-diphenyltetrazolium bromide, *OD* optical density

To evaluate the effect of cryopreservation on PDLSC proliferation, equal amounts of cells derived from cPDLSC sheets, fPDLSC sheets, and fPDLSCs were resuspended and cultivated in α-MEM with 15% FBS for 24 h, 48 h, and 72 h before being quantified by MTT assay. The MTT OD value can be found in Table 2. The OD of the blank group is 0.13. Compared to fPDLSCs at 24 h, the PDLSCs from fresh sheets had a similar proliferation rate. The proliferation rate of PDLSCs from cryopreserved sheets appeared to be slightly slower than that of the freshly prepared sheets and fPDLSCs in the first 24 h of cultivation (Fig. 3). However, no significant difference in the absorbance was observed at both 48 h and 72 h among the three groups (*P* > 0.05; Fig. 3). Overall, the experimental data indicated that neither the sheet form nor the cryopreservation and the subsequent thawing procedures suppressed the proliferative capabilities of the PDLSCs.

**Table 2:** MTT value (mean ± standard deviation)

	24 h	48 h	72 h
fresh PDLSCs	0.46 ± 0.04	0.49 ± 0.04	0.56 ± 0.03
fPDLSC sheet	0.45 ± 0.03	0.50 ± 0.03	0.53 ± 0.03
cPDLSC sheet	0.40 ± 0.05	0.48 ± 0.04	0.53 ± 0.04

Values are shown as the mean ± standard deviation

*cPDLSC* cryopreserved periodontal ligament stem cell, *fPDLSC* freshly prepared periodontal ligament stem cell, *MTT* 3-(4,5-dimethylthiazol-2-yl)-2,5-diphenyltetrazolium bromide, *PDLSC* periodontal ligament stem cell

### cPDLSC sheets maintained their multilineage differentiation potential

**Fig. 4:**
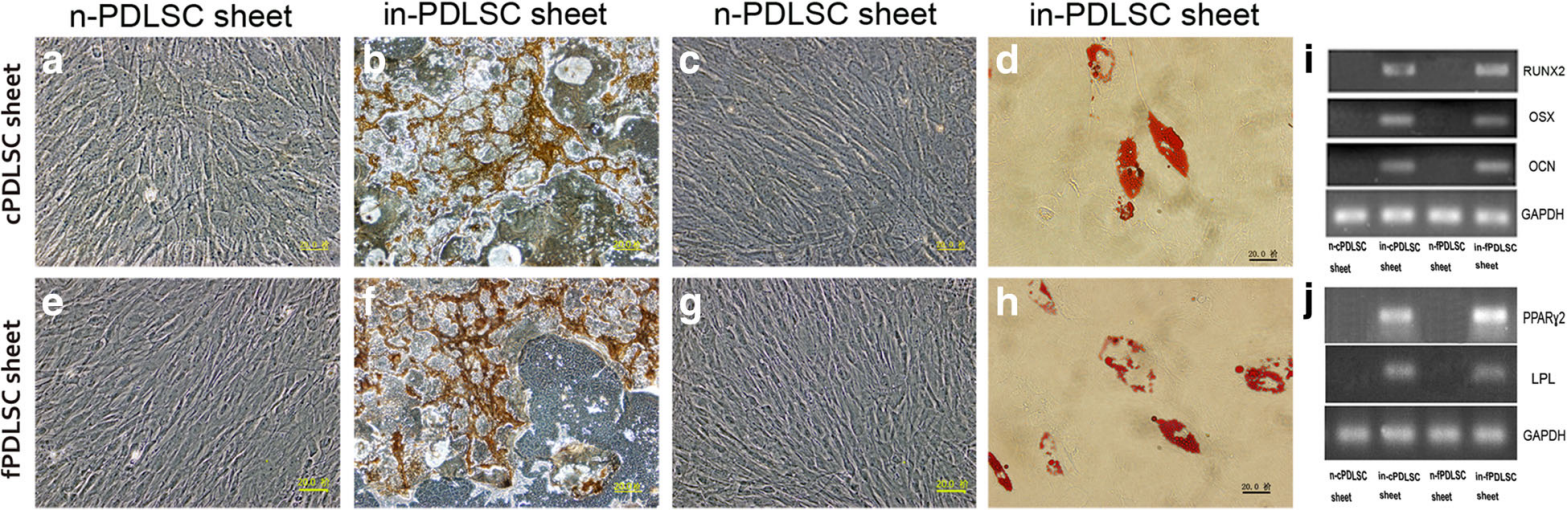
Cryopreserved periodontal ligament stem cell (*cPDLSC*) sheets retained the ability to differentiate into osteoblasts and adipocytes. Von Kossa staining showed no calcium nodules in non-induced cPDLSC sheets (**a**) and freshly prepared periodontal ligament stem cell (*fPDLSC*; **e**) sheets. Von Kossa staining showed similar calcium levels in cPDLSC (**b**) and fPDLSC (**f**) sheets. Oil Red O staining showed similar levels of lipid accumulation in non-induced cPDLSC sheets (**c**) and fPDLSC (**g**) sheets. Oil Red O staining showed similar levels of lipid accumulation in induced cPDLSC (**d**) and fPDLSC (**h**) sheets. **i** RT-PCR quantitation of runt-related transcription factor 2 (*RUNX2*), osterix (*OSX*), and osteocalcin (*OCN*) gene expression levels in PDLSCs derived from cryopreserved and freshly prepared cell sheets. **j** RT-PCR quantitation of peroxisome proliferating activated receptor γ (*PPARγ2*) and lipoprotein lipase (*LPL*) gene expression levels in PDLSCs derived from cryopreserved and freshly prepared cell sheets. *Scale bars* = 20 μm. Magnification: 40×. *GAPDH* glyceraldehyde 3-phosphate dehydrogenase, *in* induced, *n* non-induced

We next determined whether there was any difference between the cPDLSC sheets and fPDLSC sheets in regards to their multilineage differentiation potentials. On the one hand, both types of cell sheets were cultivated for 3 weeks in an osteo-inductive medium, leading to marked proliferation and the generation of dense extracellular matrices. PDLSC osteogenesis was characterized by the formation of mineralized nodules that could be identified with von Kossa staining, which offered strong evidence of calcium accumulation (Fig. 4b and f). In comparison, cell sheets that were incubated in non-osteo-inductive medium exhibited no formation of mineralized nodules (Fig. 4a and e). Osteogenic differentiation of the induced PDLSCs was further confirmed by total RNA extraction and RT-PCR detection of key osteogenesis-related regulator and effector genes, including RUNX2, OSX, and OCN. As illustrated in Fig. 4i, no significant difference was detected in gene expression levels between the cPDLSC sheet group and the fPDLSC sheet group. On the other hand, the adipogenic potentials of fPDLSC and cPDLSC sheets were determined by Oil Red O staining. The results showed that both types of cell sheets could differentiate into lipid-laden adipocytes following 3 weeks of cultivation in an adipo-inductive medium (Fig. 4d and h). Again, no adipocytes were observed when non-adipo-inductive medium was used in substitution for the adipogenic induction medium (Fig. 4c and g). RT-PCR analysis also found no significant difference between the two groups in the expression levels of PPARγ2 and LPL, both of which are adipogenesis-related markers (Fig. 4j). Taken together, the findings demonstrated that the cPDLSC sheets retained multilineage differentiation capacities on levels comparable to those of the fPDLSC sheets. Furthermore, ten fields were observed in order to calculate the number of adipocytes. There was no significant difference in the average number of adipocytes between the two groups through statistical analysis (*P* > 0.05).

### Cryopreserved PDLSCs show no alterations of karyotype

**Fig. 5:**
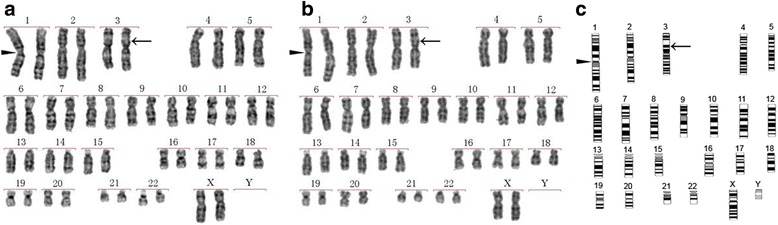
Karyograms of representative PDLSCs from cryopreserved (**a**) and freshly prepared (**b**) cell sheets. The kinetochore position (*solid triangle*), G-band position (*arrows*), chromosome length, and chromosome number were similar to the standard karyograms pattern according to ISCN 2005 (**c**)

To determine whether cryopreservation would cause chromosomal abnormalities in PDLSCs, G-banded karyotype analysis was performed. Well-spread homologous chromosomes during the mitosis metaphase were paired and arranged in the order of decreasing lengths to construct a karyogram. As shown in Fig. 5a, the cells derived from cPDLSC sheets remained diploid, with the correct chromosome number of 46. Moreover, no obvious morphological or structural aberrations, including shifts in kinetochore position (solid triangle), G-band position (arrows), or chromosome length, were observed in comparison with the PDLSCs prepared from the fresh cell sheets (Fig. 5b). They both were similar to the pattern according to ISCN 2005 (Fig. 5c). The results demonstrated that the karyotype of the cPDLSCs had no obvious karyotypic re-arrangement compared to fPDLSCs.

### cPDLSC sheets regenerated tissue in nude mice

**Fig. 6:**
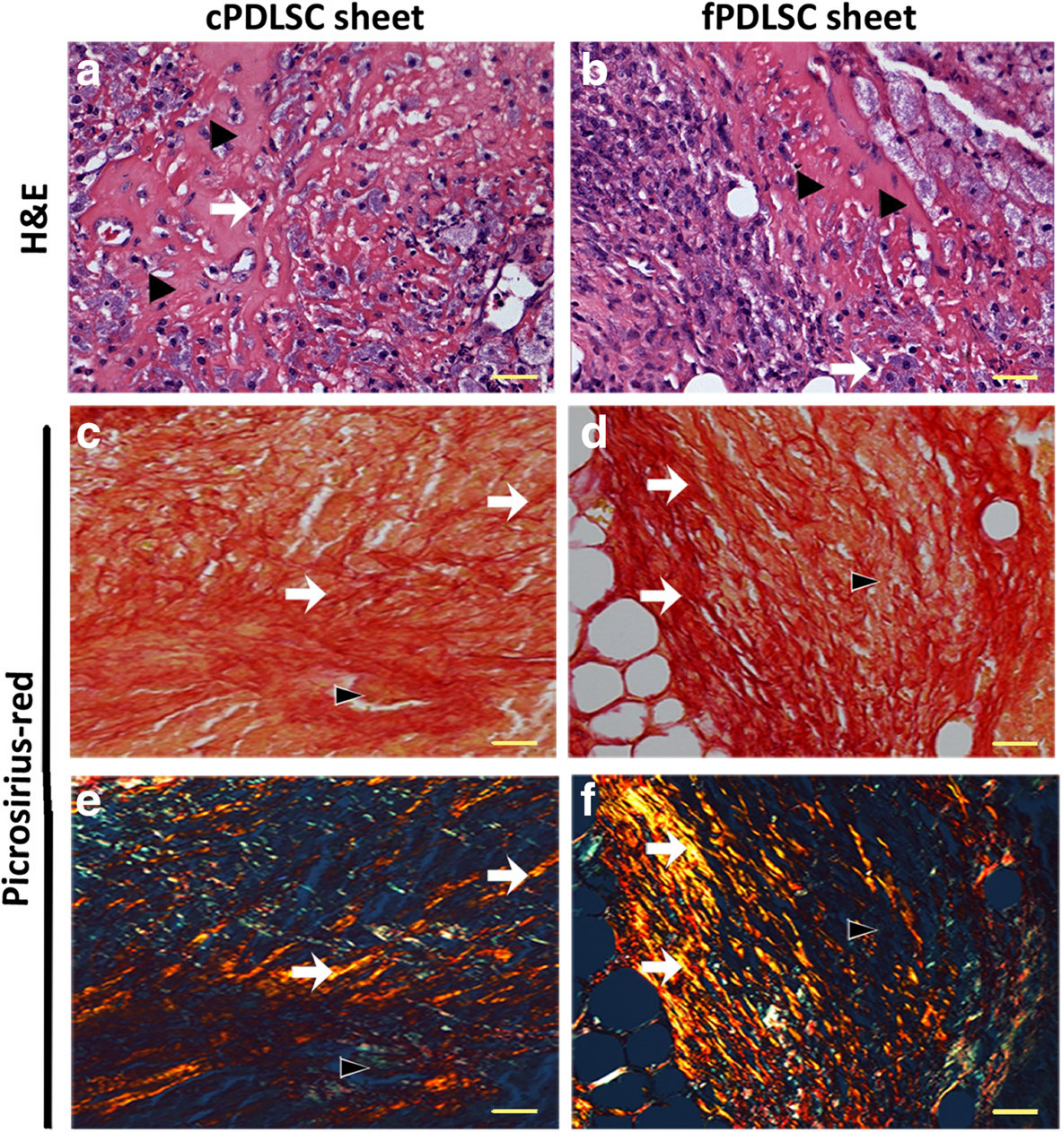
Transplated cryopreserved periodontal ligament stem cell (*cPDLSC*) sheets could develop into periodontal tissues with a well-structured ECM in nude mice. Hematoxylin and eosin (*H&E*) staining showed odontoblast-like cells (*arrows*) and bone-like matrix (*solid triangles*) developed from transplanted cPDLSC sheets (**a**) and freshly prepared periodontal ligament stem cell (*fPDLSC*; **b**) sheets. Sirius Red staining of the developed tissues showing a network of collagen type I and III proteins in cPDLSC (**c**) sheets and fPDLSC sheets (**d**). Visualization of the Sirius Red staining results in polarized light; collagen I and III are indicated in *red* (*arrows*) and *green* (*solid triangles*), respectively, in cPDLSC sheets (**e**) and fPDLSC sheets (**f**). *Scale bars* = 20 μm. Magnification: 40×

We generated a mouse model to examine the clinical utility of cPDLSC sheets for tissue regeneration in vivo. Following the subcutaneous transplantation in their dorsal regions, the recipient mice were euthanized after 4 weeks and the regenerated tissue samples were harvested for histological analysis. H&E staining revealed that the odontoblast-like cells (Fig. 6a, indicated by the arrows) and bone-like matrix (Fig. 6a, indicated by the solid triangles) grown from the cPDLSC sheets are morphologically similar to those from the fPDLSC sheets (Fig. 6b). A similar observation was obtained by Sirius Red staining, in which a network of collagen type I (in red) and type III (in green) was clearly visible under both transmitted light (Fig. 6c and d) and polarized light (Fig. 6e and f). These combined results support the notion that the cPDLSC sheets were equally effective in regenerating viable periodontal tissues as their freshly prepared counterparts.

## Discussion

Our investigation found that cPDLSC sheets can preserve PDLSC architecture similar to fPDLSC sheets in terms of cell proliferation rate, cell viability, karyotype analysis, and multilineage differentiation potential, which is set to herald the coming of cell-sheet products entering the clinical arena.

In order to obtain many cell-sheet products, cryopreservation takes on an important part of the process. DMSO [26] is one of the most commonly used cryoprotectants to aid in the long-term storage of viable biomaterials due to its ability to penetrate the cell membrane and reduce the formation of ice crystals during the freezing process [27]. There is also evidence that the electrostatic interaction between DMSO and the phospholipid components of the cell membrane is an important contributor to the cryoprotective effects of the former [28]. However, similar to many other cryoprotective agents, DMSO can exhibit cytotoxicity, particularly at a high concentration over 40% (5.1 M) [29–31]. It was shown that the use of 1–1.5 M DMSO provided the optimal protection in the cryopreservation of both dental pulp stem cells and tissues [32]. This finding was consistent with the choice of Kaku et al. of 10% (1.3 M) DMSO as the cryoprotectant for the long-term storage of whole teeth at –150 °C [16]. Based on these results, we prepared a cryopreservation solution consisting of 90% FBS and 10% DMSO to minimize cell damage and loss of viability during freezing. To our gratification, the cryopreserved cell sheets did not show any noticeable disruption of the ECM structure or loss of viability and proliferative potential. Consistent with our findings, Kaku et al. [16] and Vasconcelos et al. [20] showed that long-term storage of periodontal ligament cells and cryopreserved periodontal ligament-derived undifferentiated mesenchymal cells in 10% (1.3 M) DMSO did not negatively affect their in vitro proliferative capacities or cell viability. These results demonstrated the feasibility of cryopreservation as a potential solution to the long-term storage of PDLSC sheets.

The proliferative capacity of pre-differentiated stem cells is widely employed to predict the clinical outcome in patients receiving tissue and/or cell sheet transplant. In the current study, MTT assay was used to compare the proliferation rates of PDLSCs derived from cryopreserved cell sheets, freshly prepared sheets, and fPDLSCs. Interestingly, although no statistically significant difference was observed among the three groups of stem cells at 48 h and 72 h following their initial inoculation in the growth medium, PDLSCs that had undergone cryopreservation were found to proliferate at a slightly slower rate than the freshly prepared PDLSCs during the first 24 h. It is noteworthy that a similar delay was detected in the periodontal regeneration of cryopreserved molar transplants during a 4-week period after the operation [33]. Taken together, we demonstrated that, although PDLSCs freshly exiting the freezing stage require a period of adaptation to become sufficiently metabolically competent for growth, they retained their proliferative potentials once fully recovered.

Multilineage differentiation potential is one of the distinguishing features of mesenchymal stem cells. Although cryopreservation of adipose-derived stem cells [34, 35] and bone marrow-derived mesenchymal stem cells [36] has been achieved without the loss or alteration of their multipotent properties, no investigation has been conducted on PDLSCs. Based on our preliminary findings [37] and current study, the ability of cPDLSC sheets and fPDLSC sheets to differentiate into osteoblasts was demonstrated by both von Kossa staining and the detection of several osteogenesis-related regulator and effector genes, including RUNX2, OSX, and OCN. On the other hand, when induced in an adipogenic induction medium, both types of cell sheet were also shown to readily undergo adipogenesis. Oil red O staining and the detection of several adipogenesis-related regulator and effector genes, such as PPARγ2 and LPL, confirmed similar levels of intracellular triglyceride accumulation in the differentiated adipocytes derived from cPDLSC sheets and fPDLSC sheets. Furthermore, no significant difference in the expression of key lipogenic genes was observed by RT-PCR. Therefore, the experimental findings suggested that cryopreserved PDLSC sheets were able to maintain their ability to differentiate into multiple lineages.

Karyotype analysis is frequently performed on cryopreserved tissues to assess the impact of freezing on chromosomal stability and integrity, features that are often associated with oncogenesis [38]. Our current study found no obvious karyotypic re-arrangement for the cPDLSC sheets, which was consistent with the report of Ding et al. that cryopreserved stem cells from apical papilla retained the normal karyotype [39]. Similarly, Imaizumi et al. [40] and López et al. [41] both observed no chromosomal structural abnormalities in cryopreserved human induced pluripotent stem cells and adipose-derived stem cells, respectively. Recently, it was shown that the differentiation potency of induced pluripotent stem cells was correlated with their telomere length [42]. These findings, coupled with our experiment data, offer convincing evidence that our cPDLSC sheets could maintain similar differentiation capacity as fPDLSC sheets.

Cell-ECM interactions play a critical role in tissue regeneration by producing extracellular signals that can stimulate cell proliferation and matrix remodeling [43]. ECMs have also been established to be able to directly interact with a wide range of cell surface receptors and soluble factors through which various aspects of cellular activities are tightly modulated [44]. In our current study, the cPDLSC sheets were shown to be able to preserve ECM and its components, including fibronectin, type I collagen, and integrin, etc., in the absence of additional matrix or support. After 4 weeks being transplanted subcutaneously into the dorsal site of nude mice, the transplanted cPDLSC sheets formed bone-like matrix which exhibited a dense and interconnected network of collagen I and III, similar to that observed in tissues generated from freshly prepared cell sheets (Fig. 6). Taken together, cPDLSC sheets can be a feasible alternative for fPDLSC sheets, with an opportunity to use them in tissue regeneration.

## Conclusions

Our combined experimental data indicated that cryopreservation maintained the structural integrity and functional viability of PDLSC sheets. Therefore, the cryopreservation method, coupled with the Vc-based induction protocol that we have previously developed for cell sheet generation, could potentially serve as a solution for tissue regeneration. Further research is needed to better understand the long-term clinical effect of our cell-sheet engineering method.

## Abbreviations

BSA: Bovine serum albumin
cPDLSC: Cryopreservation periodontal ligament stem cell
DMSO: Dimethyl sulfoxide
ECM: Extracellular matrix
FBS: Fetal bovine serum
FITC: Fluorescein isothiocyanate
fPDLSC: Freshly prepared periodontal ligament stem cell
H&E: Hematoxylin and eosin
LPL: Lipoprotein lipase
MTT: 3-(4,5-Dimethylthiazol-2-yl)-2,5-diphenyltetrazolium bromide
OCN: Osteocalcin
OD: Optical density
OSX: Osterix
PBS: Phosphate-buffered saline
PDLSC: Periodontal ligament stem cell
PPARγ2: Peroxisome proliferating activated receptor γ
RT-PCR: Reverse-transcription polymerase chain reaction
RUNX2: Runt-related transcription factor 2
TBS-T: Tris-buffered saline with 0.4% Triton X-100
Vc: Vitamin C
α-MEM: Alpha-modified Eagle’s medium
